# Interaction between pedestrians and automated vehicles: Perceived safety of yielding behaviors and benefits of an external human–machine interface for elderly people

**DOI:** 10.3389/fpsyg.2022.1021656

**Published:** 2022-11-10

**Authors:** Thierry Bellet, Sébastien Laurent, Jean-Charles Bornard, Isabelle Hoang, Bertrand Richard

**Affiliations:** ^1^Laboratory of Ergonomics and Cognitive Sciences Applied to Transport (LESCOT), University Gustave Eiffel, Bron, France; ^2^ESI Group, Lyon, France

**Keywords:** automated vehicle, interaction with pedestrians, road crossing, perceived safety of AV yielding behavior, external HMI

## Abstract

This study focuses on Automated Vehicles (AVs) interactions with pedestrians during road crossing situations. A dual-phase experiment was designed: one from the pedestrian’s perspective and the other one from the AV passenger’s point of view. Eight AV behaviors to yield were investigated. Participants’ task was to assess the safety of each one of these yielding behaviors. Moreover, an external HMI (eHMI) was designed to support them in these interactions. 40 participants were involved in this experiment (50% females, 20 young versus 20 elderly). Results obtained show significant differences between old and young participants: elderly people have not the same way to perceive and assess the safety of the yielding behaviors from “the inside” and from “the outside” of the car. Conversely, young participants assessed AV behaviors similarly whether as pedestrians or as AV passengers. When considering benefits introduced by the eHMI, it significantly reduces differences between old and young participants and tends to harmonize their safety assessments: with to the eHMI, elderly people are more able to adequately perceive and assess the safety/dangerousness of the AV braking manoeuvers, and their safety judgments become at last quite similar to those of young participants. Moreover, the eHMI increases participants’ Acceptance of AV and reduces their concerns about their future interactions with AV as a pedestrian, especially for elderly people.

## Introduction

With the development of Automated Vehicles (AVs), interactions between cars and pedestrians will soon become a challenging issue for road safety. This study aims to anticipate these future interactions in road-crossing situations. Indeed, when introduced on public roads, AVs will coexist with conventional vehicles as well as with pedestrians. In order to contribute to a safe traffic system and to ensure the public acceptance of AVs, the one key challenge is to study how pedestrians will perceive and assess the AVs’ yielding behaviors when they are waiting on the pavement with the willingness to cross a road.

To achieve safe interactions, pedestrians and drivers need to share a common situation awareness (SA) of traffic situation. SA was defined by Endsley (1995) as “*the perception of the elements in the environment within a volume of time and space, the comprehension of their meaning, and the projection of their status in the near future*.” As a mental representation of traffic situation, SA plays a key role for risk assessment and decision-making when interacting with other road users (Bellet et al., 2009). In case of misinterpretation of others’ intentions, critical accidents involving pedestrians may occur (Habibovic and Davidsson, 2012). The Fatality Analysis Reporting System of the National Highway Traffic Safety Administration, NHTSA (2017), revealed that more than 25% of pedestrians’ fatal crashes were caused by a lack of communication and mistaken assumptions about others’ actions. Street-crossing situations involve a complex decision-making process based on several factors (Lévêque et al., 2020). In this context, and especially when ambiguities remain regarding priority rules (i.e., non-signalized crossing), pedestrians and car drivers tend to interact using non-verbal communication (e.g., facial expressions, eye contacts, gestures, and body movements) in order to clarify their intentions. The importance of such communication to ensure roadway safety has been widely documented in the field of traffic psychology. For instance, Schmidt and Färber (2009) discussed the role of eye contact between pedestrians and drivers. They showed that pedestrians willing to cross a street usually look at the approaching vehicle, to make sure the driver sees them. Once their eye contact is returned, pedestrians suppose they were noticed by the driver, and that mutual understanding was achieved. From a study carried out with a large sample of pedestrian behaviors, similar conclusions were reached by Rasouli et al. (2017) who found that, in the context of non-signalized crossings, more than 90% of pedestrians gazed at oncoming vehicles before crossing. Although recent studies have expressed some reservations about the exact role of eye contacts (Dey and Terken, 2016; Al Adawy et al., 2019; Lee et al., 2021), non-verbal communication (such as head movements or hand gestures of car drivers, for example) may play a key role to support pedestrians’ decision-making in ambiguous situations, and then to increase their safety.

Therefore, a key concern regarding the introduction of AVs on public roads is that they may negatively impact interactions with pedestrians because of the changing status of the drivers. With control transferred to automation, drivers can be involved in secondary tasks, and pedestrians will not be able to rely on cues from their behaviors anymore. This could lead to misinterpretation of the AV’s intention and increase the risk of critical conflicts. In their study, Malmsten Lundgren et al. (2016) suggested that the introduction of AVs in the urban context may lead to notable change in how pedestrians experience AVs compared to conventional vehicles. Pedestrians reckoned that non-verbal communication with the driver promoted a safe interaction, whereas apparent driver distraction in the AV (e.g., phoning or reading the newspaper) increased pedestrians’ stress and was associated with an unpleasant interaction.

Moreover, beyond the stress experienced, a new set of road safety issues also arise according to the behavioral difference between automated versus conventional vehicles piloted by real humans, specifically regarding older people. Indeed, the literature shows that elderly people have more difficulties than younger pedestrians to identify time gap and make a safe decision to cross, particularly in the frame of complex traffic situations or when facing a continuous flow of approaching vehicles (Lobjois and Cavallo, 2007; Schmidt and Färber, 2009; Dommes, 2019; Núñez Velasco et al., 2019). Speeds of approaching vehicles were also identified as particularly important risk factors for elderly pedestrians, who may have difficulties to perceive and to adequately estimate them before implementing their road-crossing (Cavallo et al., 2009; Beggiato et al., 2017). At this level, the aim of this experiment will be to study if they perceive and assess the safety of different AV’s braking behaviors to yield in the same way than young participants or, as assumed in the literature, if they have specific age-related difficulties in this safety evaluation task.

One possible solution to manage this risk and to facilitate interactions and negotiations between AVs and pedestrians is to equip AV with an external Human–Machine Interface (eHMI). Such an eHMI may be used as an explicit way to communicate (i.e., able to reinforce implicit cues related to AV behaviors) about AV’s status and intention to yield through pictograms, text messages, or lights (Beggiato et al., 2017; Habibovic et al., 2018; Ackermann et al., 2019; Bazilinskyy et al., 2019; Schieben et al., 2019; see Dey et al. (2020) for a classification taxonomy of existing eHMI). In the frame of this study, such an eHMI was designed to support AV’s interactions with other road users, and then was evaluated from both the AV passenger and the pedestrian’s point of view.

In this general context, our main research questions are the following: How pedestrians will perceive and assess the safety of AV behaviors to yield before road crossing? Which kind of braking behavior will be perceived as safe or not, according to the AV deceleration and stopping distance? Will these safety assessments be similar when the participant will experience the interaction as a pedestrian, or as an AV passenger? How will elderly people perceive and assess the safety of AV’s behaviors to yield compared to a group of young participants? And finally, regarding the designed eHMI, to which extent will it reduce pedestrians’ worries and increase their acceptance of AVs?

## Materials and methods

**Figure 1:**
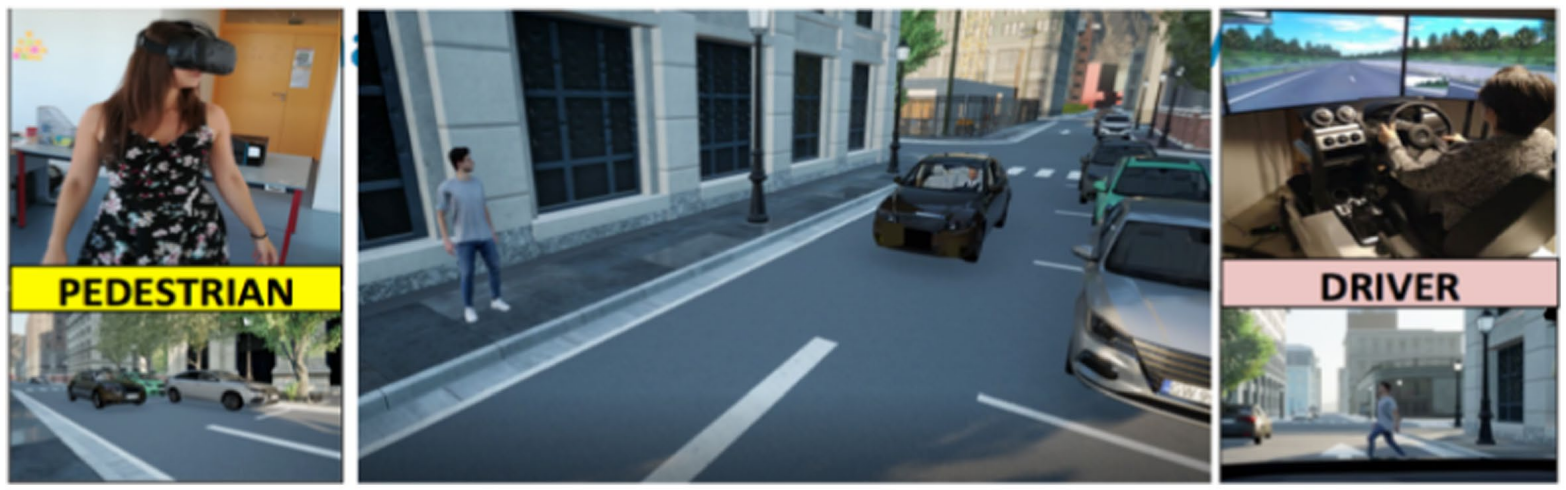
Pedestrian and automated vehicle scenario of interaction.

In order to study how interactions between pedestrians and automated vehicles may look like in the future, and how road safety may be affected by the AV behaviors, an immersive experiment was implemented to allow real humans to « practically experience » (even if in a virtual way) future interactions with such AVs. With this aim, a set of driving scenarios was developed with to the *Virtual Human-Centred Design* platform (i.e., *V-HCD*; Bellet et al., 2019). Figure 1 presents the urban environment developed for this experiment, at the moment of the driving scenario where the pedestrian is willing to cross, while the AV is approaching.

To allow participants to experience immersive interactions with automated vehicles both from the outside and on-board the AV, a dual-phase experiment was designed: one phase focused on the pedestrian’s perspective, and the other from the AV front passenger’s point of view (i.e., driver seat).

### From the pedestrian point of view

**Figure 2:**
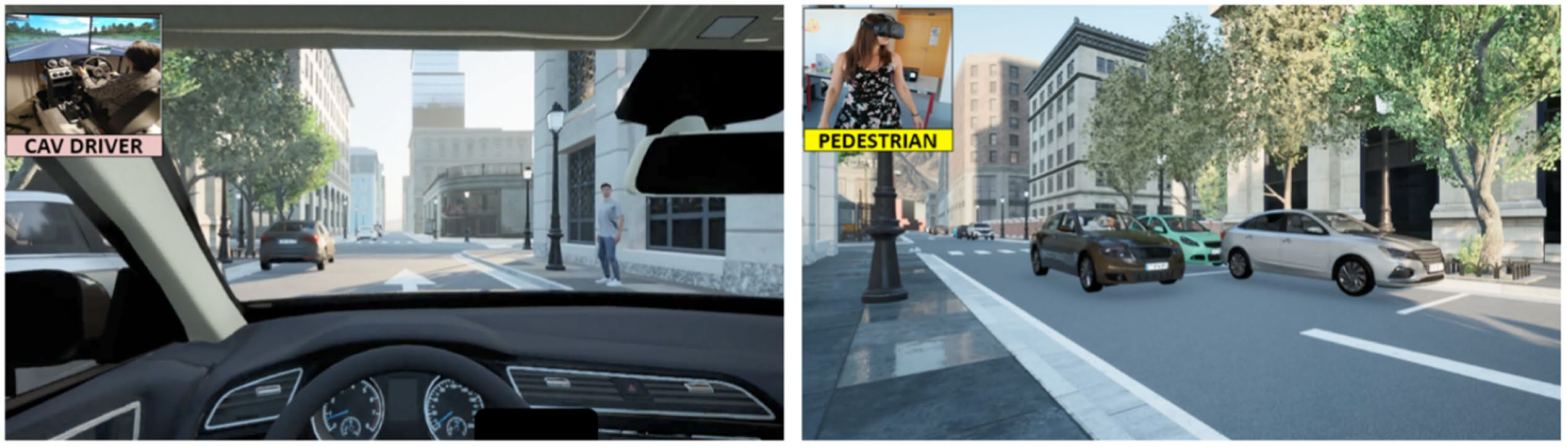
The situation as seen by the automated vehicle (AV) passenger (left) and the pedestrian (right).

To support the experiment from the pedestrian’s point of view, a virtual reality head-mounted display VIVE^™^ Pro Eye was used to simulate a realistic environment with a resolution of 1,440 × 4,600 pixels per eye, a field of view of 110 degrees, and a refresh rate of 90 Hz. Figure 2 (right view) shows the road environment as perceived by the participant as a pedestrian. This part of the experiment focuses on safety assessment of AV behaviors to yield, without effectively implementing the crossing behavior. Participants are located on the pavement facing a continuous flow of approaching vehicles. First, a randomized number of vehicles (conventional and automated) do not stop, and then an AV approaches and stops according to different braking strategies. Participants have to randomly experience these scenarios and assess the safety of each AV yielding behavior.

### From the AV passenger point of view

The part of the experiment involving AV passengers was performed on a dynamic simulator (i.e., “*Develter Pro-Evolution”* cabin) to support controlled examination of interactions with simulated pedestrians. Even if the dynamics of this simulator is limited (i.e., based on four small cylinders), it is however possible to simulate different levels of braking. Figure 2 (left view) presents the road scene as seen from the AV passenger’s point of view. During this part of the experiment, as for the previous one, participants experienced randomly eight AV braking strategies when yielding to a pedestrian.

### Automated vehicle yielding behavior

**Figure 3:**
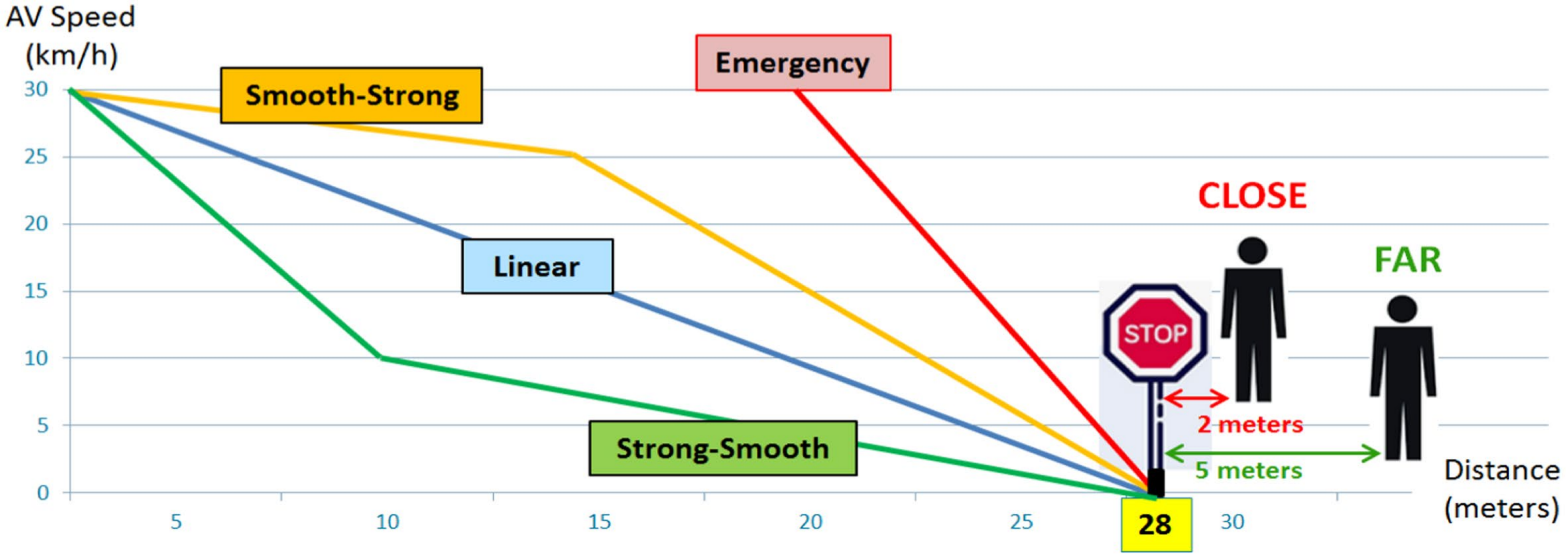
Braking behaviors to yield of the automated vehicle.

A total of eight yielding behaviors were presented to the participants for each modality studied in this experiment. These eight scenarios correspond to four decelerating behaviors (i.e., in terms of ways to brake and AV dynamics) associated with two stopping distances, as illustrated in Figure 3.

The four AV maneuvers to yield are: “*Linear”* deceleration (i.e., braking linearly on a distance of 28 meters), “*Strong-Smooth”* (i.e., a strong braking followed by a smooth deceleration; this strategy could facilitate the detection of the beginning of the AV braking maneuver by pedestrians), and “*Smooth-Strong”* (i.e., a smooth followed by a strong deceleration; this strategy could be more gradual and comfortable for the AV passenger). These three strategies were inspired by real drivers’ behaviors to yield observed in naturalistic traffic conditions in a 30 km/h urban area (Schneemann and Gohl, 2016). Conversely, the last yielding behavior, i.e., “*Emergency,”* is automation-inspired and simulates the reaction of an Automated Emergency Braking system (AEB) when facing a dangerous crossing decision of a pedestrian (i.e., very strong linear braking on a very short distance of 8 m, corresponding to the maximum braking power of a real car).

These four yielding behaviors are associated with two alternative stopping distances: “*Far*” (distance of 5 m from the pedestrian) and “*Close*” (distance of 2 m).

After each scenario, the participant (as a pedestrian or as an AV passenger) will be asked to assess the safety of the AV behavior to yield, using a continuous scale ranging from 0 (i.e., “*totally unsafe*”) to 100 (i.e., “*totally safe*”). The motivation for using such a 0–100 Likert scale in this experiment (against 5- or 7-level Likert scales, more commonly used in social sciences) was to collect individual assessments formulated through continuous numerical values, the latter being required in order to support quantitative statistical analyses based on parametric tests, like ANOVA or *t*-tests (Chimi and Russell, 2009; Yusoff and Mohd Janor, 2014; Bellet et al., 2018).^1^

As a first hypothesis, it is expected that among these eight braking behaviors, some of them should be perceived as safer than the others, more particularly from the pedestrian point of view. Typically, a Far stopping distance of 5 m should be evaluated as being safer for pedestrians than a Close 2-m distance. Moreover, the AEB behavior was designed as an emergency reaction that is not really suitable to the scenarios studied in this experiment (i.e., AV approaching to a pedestrian who is quietly waiting to cross on the pavement). Thus, because of their critical nature and their inappropriateness for our scenarios, these AEB behaviors should be perceived as being less safe than the six other AV yielding maneuvers (whether associated with a Far or a Close stopping distance). As a second hypothesis, because of the literature showing that elderly people have more difficulties than younger pedestrians to adequately assess the speed of approaching vehicles, it is assumed that, as pedestrians, old participants will have more difficulties than young to perceive the inadequacy of the AEB braking behavior.

### External HMI

**Figure 4:**
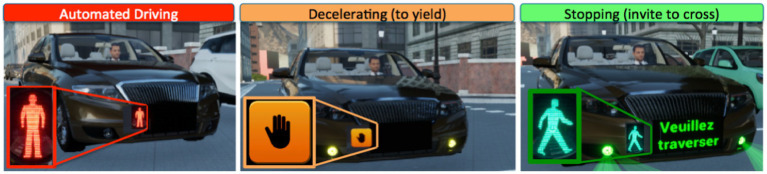
Pictograms of the external human–machine interface (HMI) used to inform pedestrians about the AV status.

The eHMI designed for this study is made of two components: a pictogram, presented on the vehicle grille to explicitly indicate the AV status, and a set of lights able to dynamically draw crosswalks on the street to communicate with pedestrian about the crossing opportunity. Figure 4 presents the 3 pictograms of the eHMI according to the AV status. During automated driving (1st image), a red pedestrian (similar to the symbol used on traffic lights) is presented, informing other road users not to cross. Then, when the AV decides to yield and starts to decelerate, a new pictogram is presented (i.e., 2nd image: a black hand in an amber square) to inform the pedestrian about the AV yielding intention, however requiring the former to wait for some additional seconds the full stop of the car before crossing. Finally, when the AV is stopped, a green pedestrian pictogram is displayed in combination with the short text “*Veuillez Traverser*” (i.e., “*Please Cross*”; *cf.* 3rd image).

**Figure 5:**
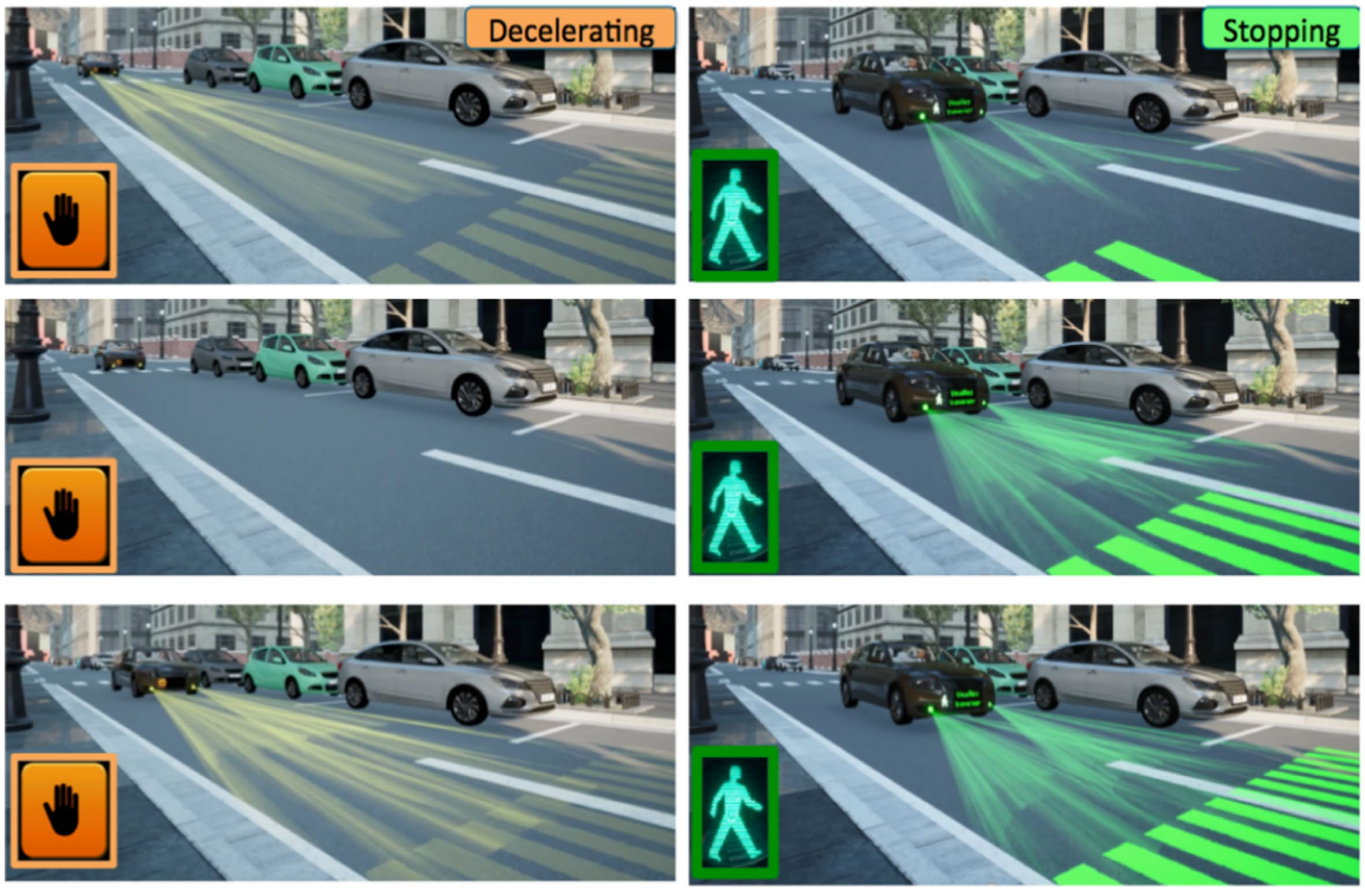
eHMI when the AV is decelerating (left sequence) and stops (right sequence).

These two pieces of information, based on existing solutions that are commonly used today to communicate with pedestrians on public roads (by traffic lights or as road signs, that are replicated in this study on the AV’s grill), were provided to facilitate the participants’ intuitive understanding of the AV status without any additional explanation. However, the key eHMI component designed to support the pedestrians’ assessment of AV’s behaviors safety is a dynamic lighting solution, presented in Figure 5, aiming to gradually communicate and inform them about the AV intentions. When the AV detects a pedestrian and makes the decision to yield, a set of flashing lights dynamically projects a blinking amber crosswalk on the street during the AV decelerating phase (i.e., left sequence on Figure 5). Then, when the AV is stopped, a green crosswalk is drawn gradually on the road to invite the pedestrian to cross (*cf.* right sequence of Figure 5).

**Figure 6:**
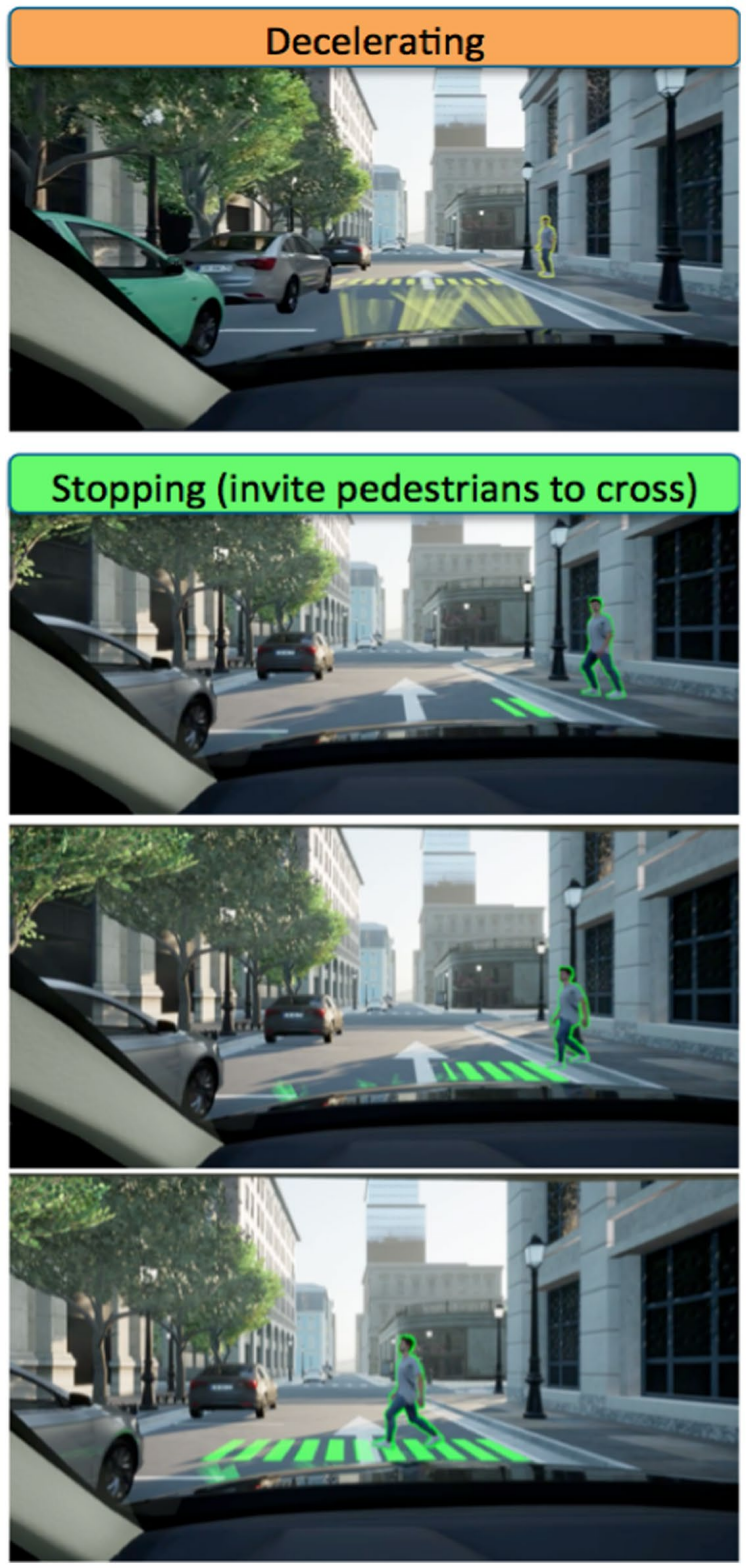
The eHMI, from the AV passenger’s point of view.

Lastly, Figure 6 presents the eHMI as perceived from the front AV passenger point of view (i.e., from the driver’s seat). In association with the external drawing of the crosswalks, an additional information is delivered through the head-up display to inform the passenger about the AV’s perception, decision, and intention, taking the form of the outline of the detected pedestrian required to be yielded (in *Amber*, during the decelerating phase, and then in *Green*, when the AV stops).

### Questionnaires: eHMI evaluation and benefit for supporting interactions with AV

After having experienced the eHMI (as a pedestrian or as an AV passenger, respectively), participants will have to complete two times a 4-item questionnaire (from 0 to 100 scales) about the “*eHMI qualities*” (in terms of its “*Usefulness*,” “*Intelligibility*” and “*Temporality*” of the delivered information) and about its benefit on their “*AV Acceptance*” (collected in a dual way: acceptance of AV without versus equipped with the eHMI).

This experiment is also taking place in the “before” versus “after use” paradigm (Distler et al., 2018) in terms of impact of the eHMI on participants’ perceived safety and worries about interactions with AVs as pedestrians. With this aim in mind, an AV safety questionnaire was completed by the participants before the experiment (*a priori* judgments), and then filled in at two additional times: (1) after having experienced the eHMI as a pedestrian and (2) as an AV passenger (or in the opposite order for half of the participants). This AV Safety questionnaire is made of 2 blocks of 3 items (collected from a set of “7-point scales,” ranging from 1 [*totally disagree*] to 7 [*totally agree*]). The 1st one is dedicated to *AV risks* (i.e., “*AVs would pose minimal risk to its driver and passengers*,” “*AVs would pose minimal risk to other road users*” and “*AVs would be safe*”), and the 2nd one focuses on *Worry to interact with an AV as a pedestrian* (i.e., “*I would cross the road in front of AVs,”* “*I would have no concerns walking as usual if AVs would be on public roads*” and “*The prospect of interacting with AVs as a pedestrian appeals to me*”). From this replication method, it will be possible to evaluate to which extent the eHMI may change participants’ perception of AVs safety and reduce their concerns toward interaction with AVs, compared to their *a priori* judgments (i.e., before having experienced it).

### Test procedure

**Figure 7:**
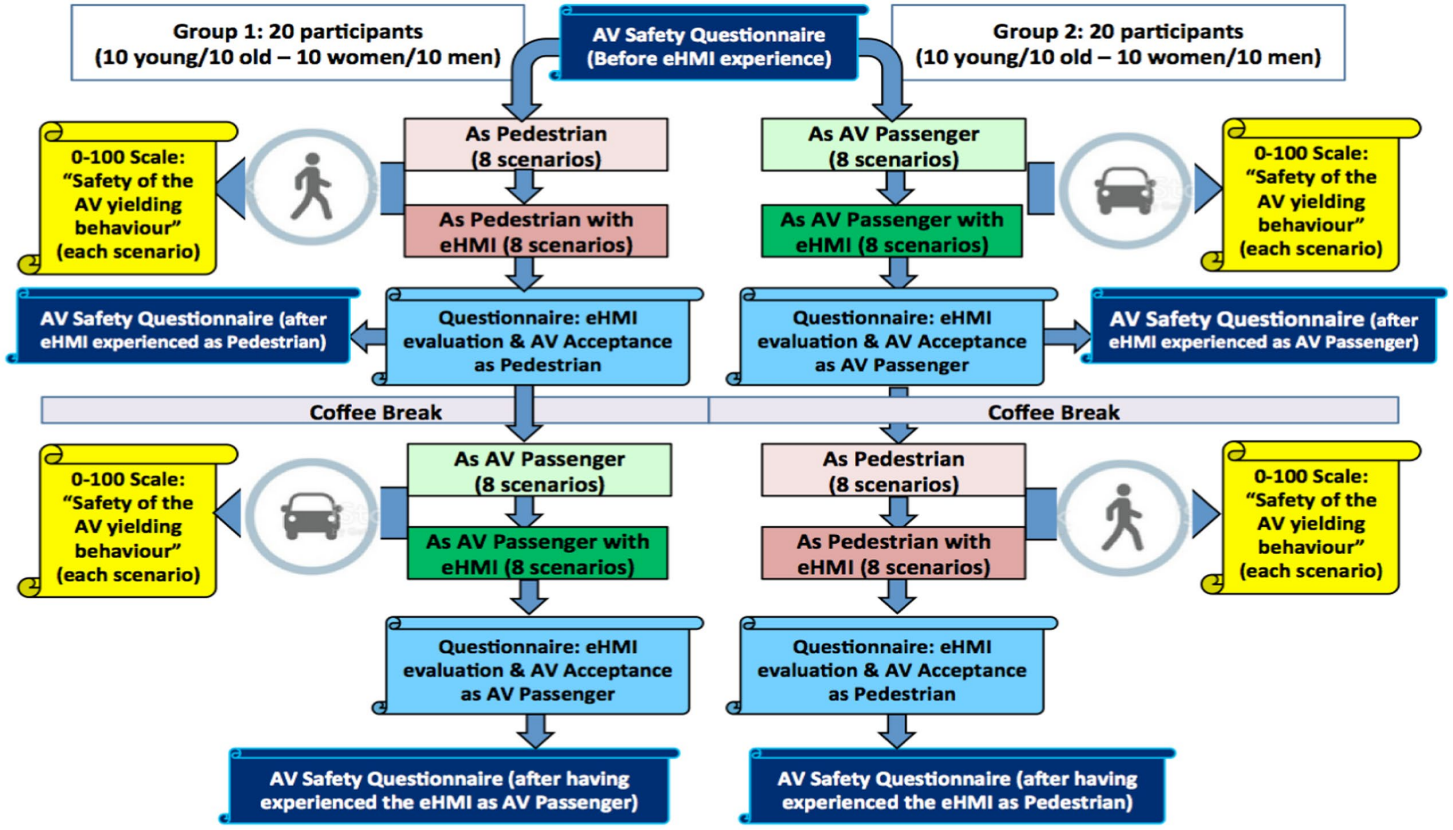
Overview of the test procedure.

Figure 7 provides an overview of the test procedure implemented for this study. Before the experiment, all the participants completed the AV Safety questionnaire (*a priori* judgments).

Then, to control potential learning effects and to familiarize the participants with the virtual environment, a training session (based on 6 scenarios) was performed before the experiment (3 as a pedestrian and 3 as an AV passenger). Moreover, two similar groups of 20 participants were built (50% females–50% males, 50% old–50% young). The first group started with the experiment from the pedestrian’s point of view (without, and then with the eHMI), and then performed the experiment from the AV passenger’s point of view (without, and then with the eHMI). The second group did the opposite. In addition, to avoid order effect inside each sub-part of the experiment, a randomized delivery strategy of the scenarios was implemented.

In total, each participant experienced 32 scenarios (8 as a pedestrian without eHMI + 8 as a pedestrian with the eHMI + 8 as an AV passenger without eHMI + 8 as an AV passenger with the eHMI). At the end of each scenario, the traffic simulation is frozen (pause) and the participant is asked to evaluate the safety of the AV yielding behavior from a continuous scale ranging from 0 (i.e., “totally unsafe”) to 100 (i.e., “totally safe”).

Finally, after having experienced the eHMI (as pedestrian or as AV passenger, depending of the group), participants completed a 2nd time the “AV Safety” and the “eHMI evaluation and AV Acceptance” questionnaires, and then a 3rd time at the end of the experiment.

### Participants

Forty participants were involved in this experiment: 20 young drivers (10 males and 10 females, mean age of 25.1 years, S.D. 4.1 years) and 20 elderly drivers (10 males and 10 females, mean age of 67.5 years, S.D. 4.5 years). All the participants have a valid driving license and were driving a car regularly.

### Instructions given to the participants

To evaluate the safety of the AV yielding behavior as an AV passenger, participants received the following instruction: “*For this experiment, you will take place on-board a simulated autonomous car piloted by the actual algorithm developed by a car manufacturer. When this AV will decide to yield a pedestrian, you will have to evaluate—from your AV passenger point of view—the safety of its braking behaviour by using a continuous scale ranging from 0 (totally unsafe) to 100 (totally safe)*.”

To evaluate the safety of the AV yielding behavior as a pedestrian, participants received the following instruction: “*For this experiment, you will take place on the pavement facing to a continuous flow of vehicles (automated or manually driven). One of them is an autonomous vehicle piloted by the actual algorithm developed by a car manufacturer. When this AV will decide to yield, you will have to evaluate—from your pedestrian point of view—the safety of its braking behaviour by using a continuous scale ranging from 0 (totally unsafe) to 100 (totally safe)*.”

## Results

As a first general result, not any significant order effect was found between the data collected for groups 1 and 2 (*t*-tests), meaning that experiencing the scenarios as a pedestrian “before” or “after” having experienced them as an AV passenger has not any significant impact on participants’ evaluations.

### Evaluations of the safety of AV yielding behaviors (without eHMI)

#### Perceived safety of the AV behaviors according to the stopping distances

**Figure 8:**
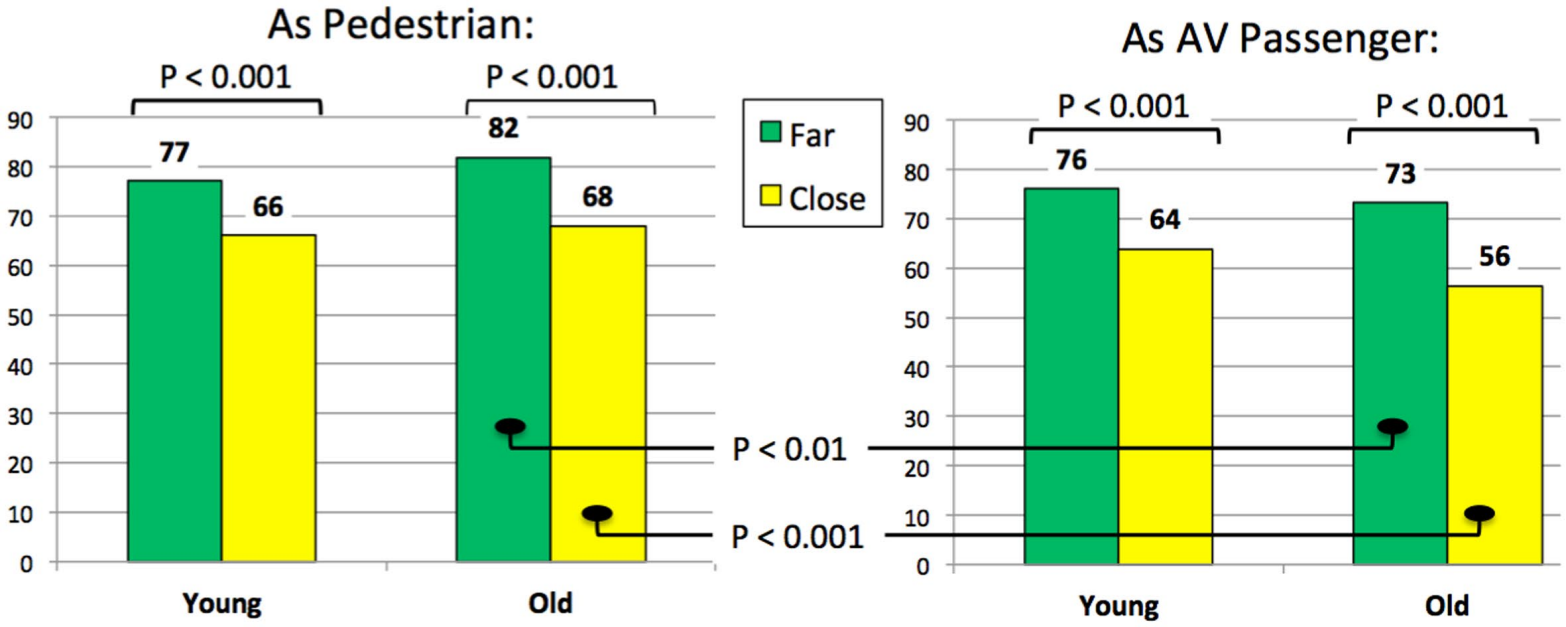
Perceived safety of the AV yielding behaviors according to the distance to stop.

Figure 8 presents participants’ safety assessments related to the AV yielding behaviors when they experienced the interaction as pedestrians (on the left) or as AV passengers (on the right). In accordance with our initial hypotheses, both old and young participants perceive the *Far* stopping distance as safer as pedestrians. Similar results are also found as AV passengers. These results are not surprising, because the more the stopping distance is far to the pedestrian, the more the safety margin is important, and the less the situation is perceived as being risked.

More interestingly, a significant difference is also found for the sample of older participants: their safety ratings about AV behaviors are significantly higher (paired *t*-tests), for both Far and Close distances, when assessed from the pedestrian than from the AV passenger point of view. This result indicates that elderly people have not the same way to perceive and assess the safety of AV yielding behaviors from “the inside” and from “the outside” of the car: as a pedestrian, they assess them significantly safer than as a passenger. Conversely, it is not the case for the young participants, who rate AV behaviors similarly, whether as pedestrians or as AV passengers.

#### Safety assessment of the AV braking maneuvers to yield

**Figure 9:**
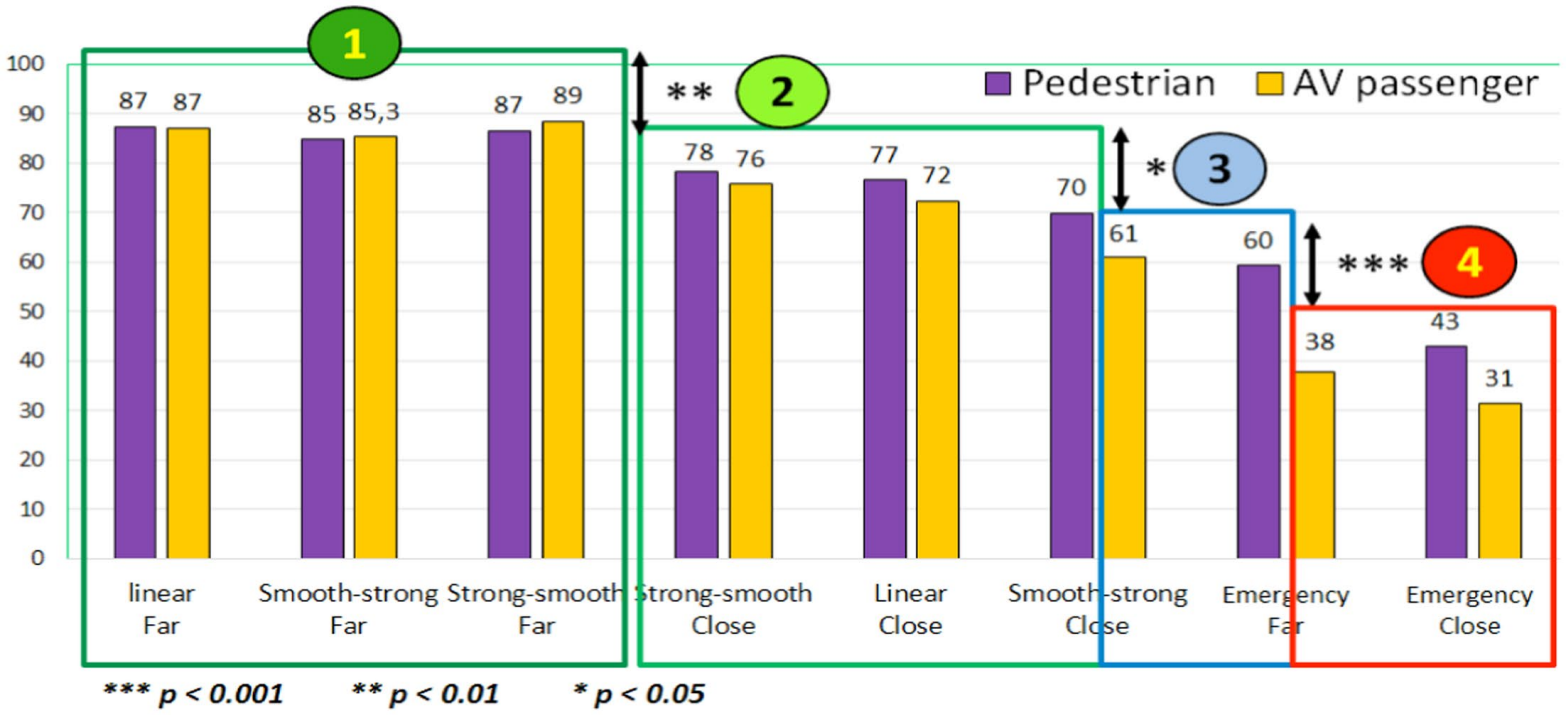
Participants’ evaluations about the safety of different AV behaviors to yield, from the pedestrian and the AV passenger point of views.

As shown in Figure 9, combining old and young participants’ ratings, significant differences (paired *t*-tests) appear between four main blocks of AV braking behaviors in terms of safety assessment, both as pedestrians and as AV passengers. As initially assumed, the Far stopping distance associated with Linear, Strong-Smooth, and Smooth-Strong deceleration is assessed as highly safe by all the participants (more than 80/100, corresponding to a high positive value; *cf.* Distler et al., 2018). At the other extremity of the string, Close Emergency braking of the AV is assessed as an unsafe behavior to yield, especially from the AV passenger’s point of view (31/100).

#### Perceived safety of AV behaviors to yield, according to participants’ age

Despite these global similarities between all the participants in terms of AV behaviors safety assessment, some local statistical differences appear between old and young.

**Figure 10:**
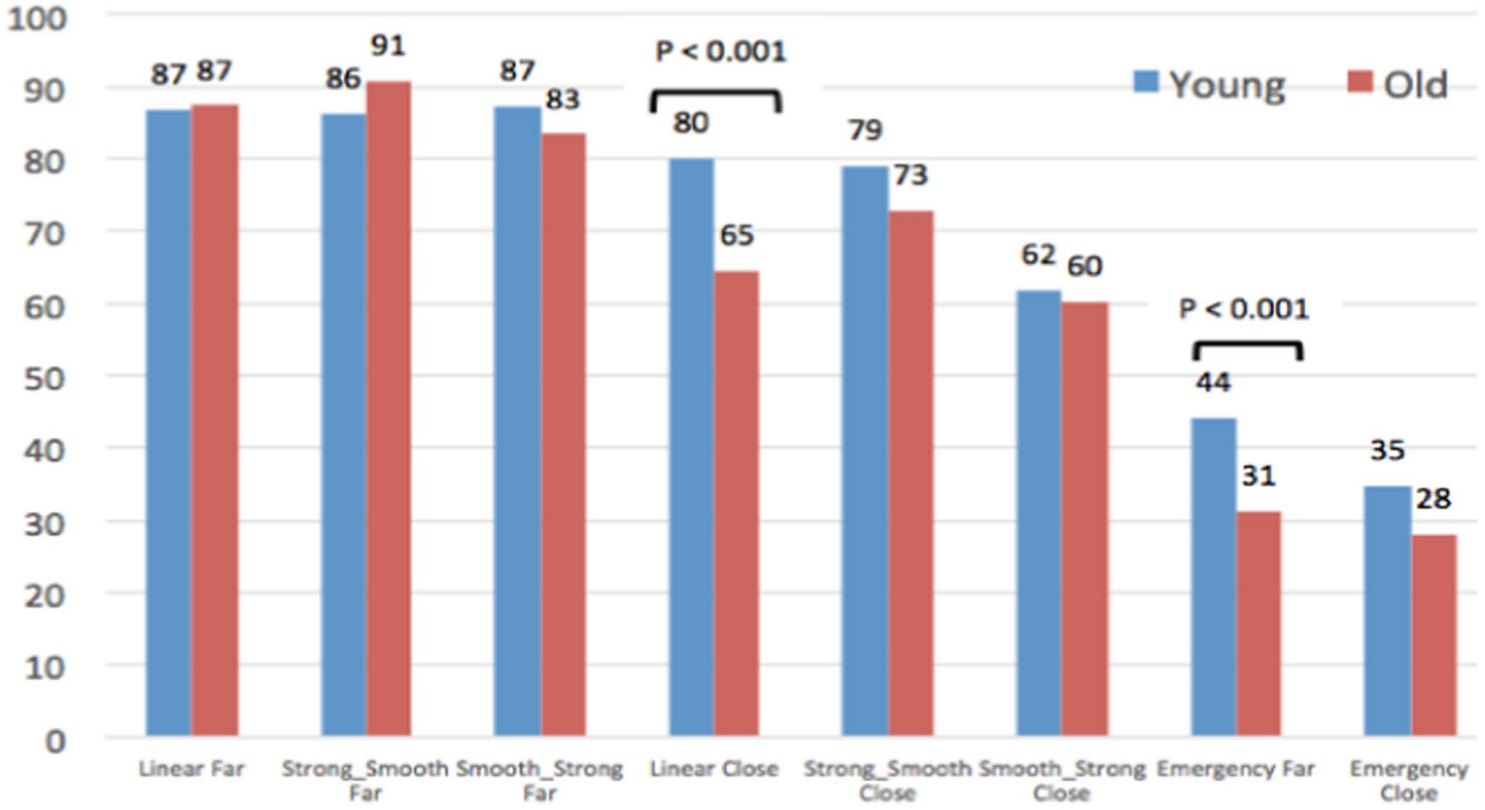
Evaluation of the safety of the AV yielding behaviors, as an AV passenger.

As AV passenger (Figure 10), two AV yielding behaviors are assessed as significantly less safe by old compared to young participants: *Linear close* and *Emergency Far*.

**Figure 11:**
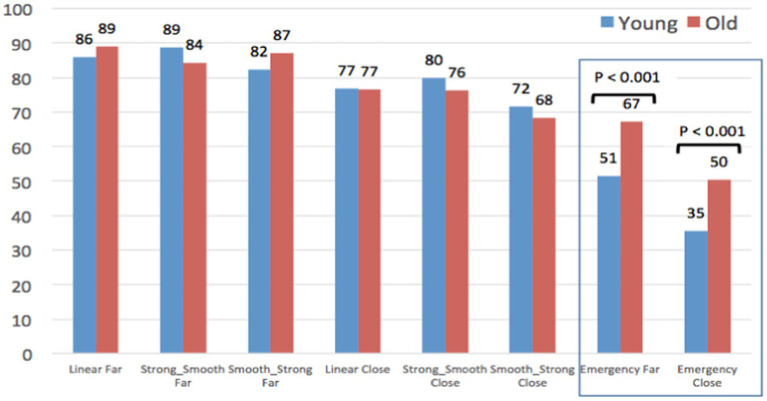
Evaluation of the safety of the AV yielding behaviors, as a pedestrian.

Conversely (Figure 11), as pedestrians, old participants assess the AEB Emergency braking as significantly safer than young participants. This result seems surprising, according to their higher vulnerability in case of accident. It is the case for Emergency Close, but also in a more impressive way for the Emergency Far yielding behavior, which is assessed as very safe by elderly people (67/100), against neutral by young participants (51/100).

Finally, when jointly considering results of Figures 10 and 11, differences between old and young participants are highly contrasted: Although young participants provide quite similar judgments from the Pedestrian and the AV Passenger’s point of views, older participants’ ratings are totally different regarding the 2 Emergency braking (difference of 36 points for Emergency Far, and of 22 points for Emergency Close). As pedestrians, it seems that elderly participants have difficulties to visually perceive and evaluate the abruptness of the AEB deceleration, which is not the case when they experience the same AEB yielding behaviors on-board the AV.

### Evaluations of the benefits introduced by the external HMI

#### External HMI benefits to perceive and assess the safety of AV behaviors to yield

**Figure 12:**
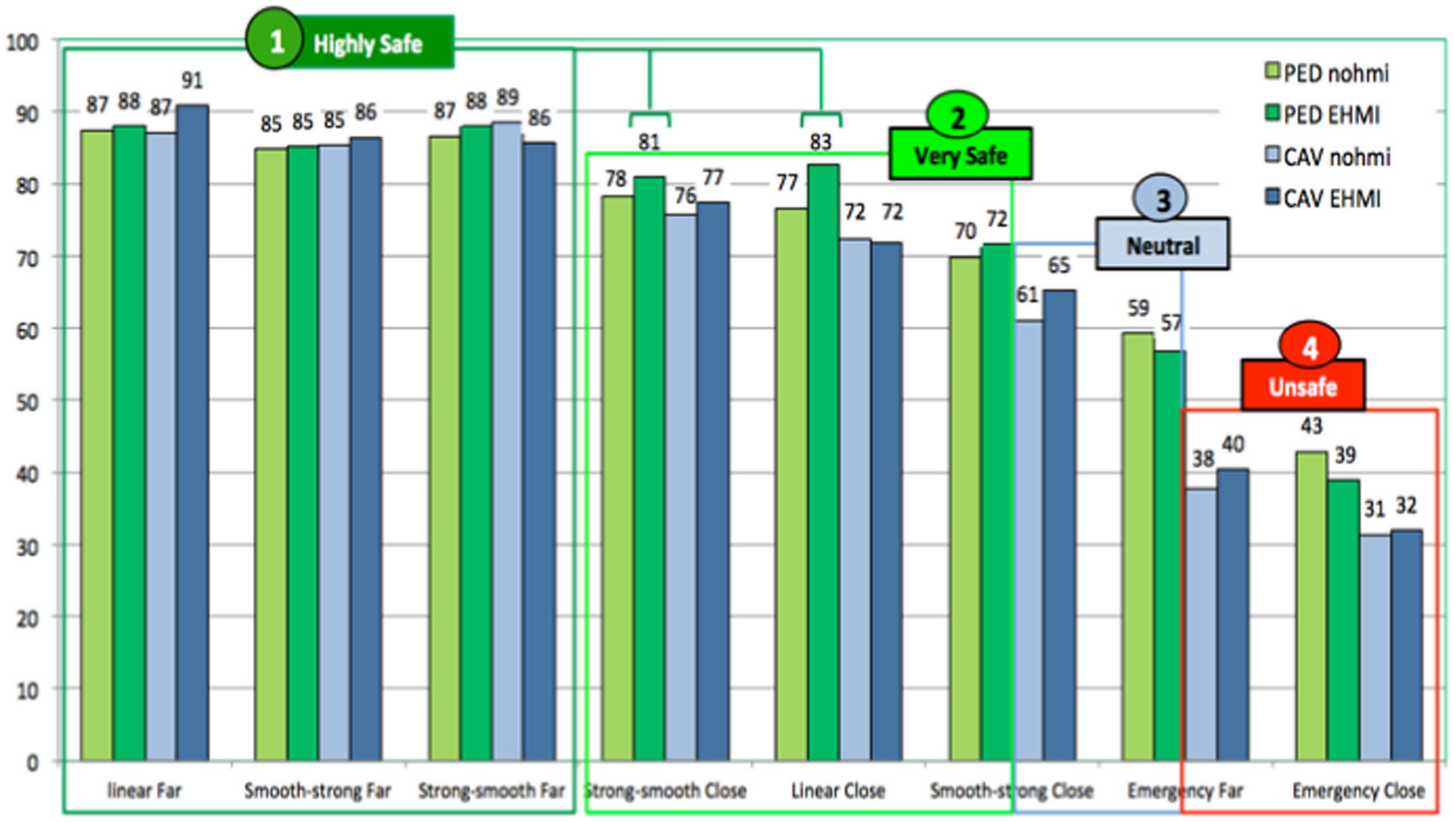
Hierarchy of AV braking behaviors safety, without and with the eHMI.

At first glance (Figure 12), the eHMI does not radically change participants’ safety judgments: Far stopping distances are perceived as being more safe than close distances, and the hierarchy between the different AV braking maneuvers is quite similar to Figure 9.

However, two interesting results must be mentioned. First, the eHMI increases pedestrians’ feeling of safety for Linear Close and Strong-Smooth Close braking, which obtain ratings over 80/100 thanks to the eHMI solution (i.e., highly safe).

**Figure 13:**
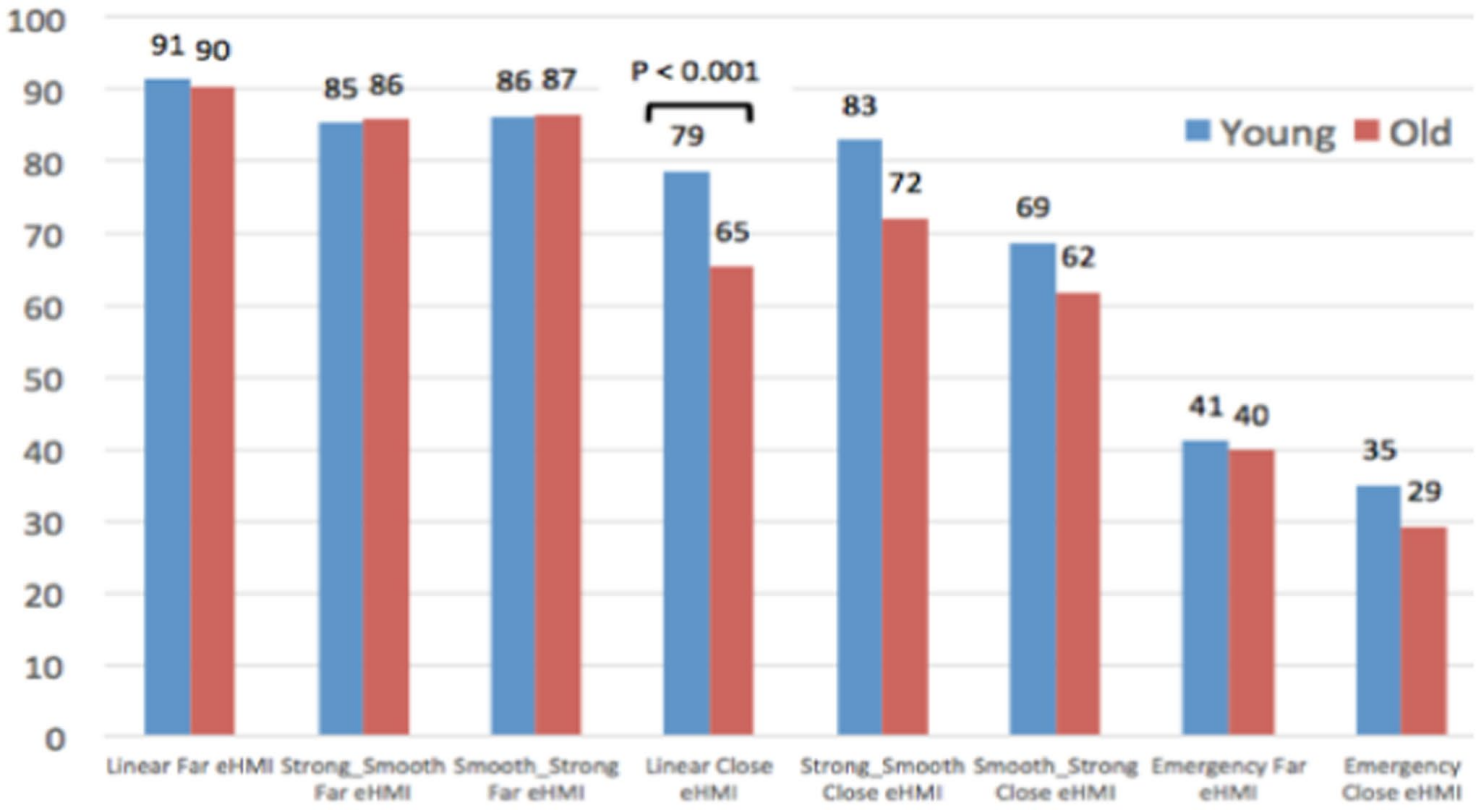
Safety assessment of AV (with eHMI) behavior to yield, as an AV passenger.

In addition (Figure 13), and interestingly, the eHMI also tends to reduce the differences previously observed between old and young participants about AEB behaviors. As AV passengers, not any significant statistical difference is found between these groups for the Emergency braking (Far and Close), which are similarly assessed as not safe.

**Figure 14:**
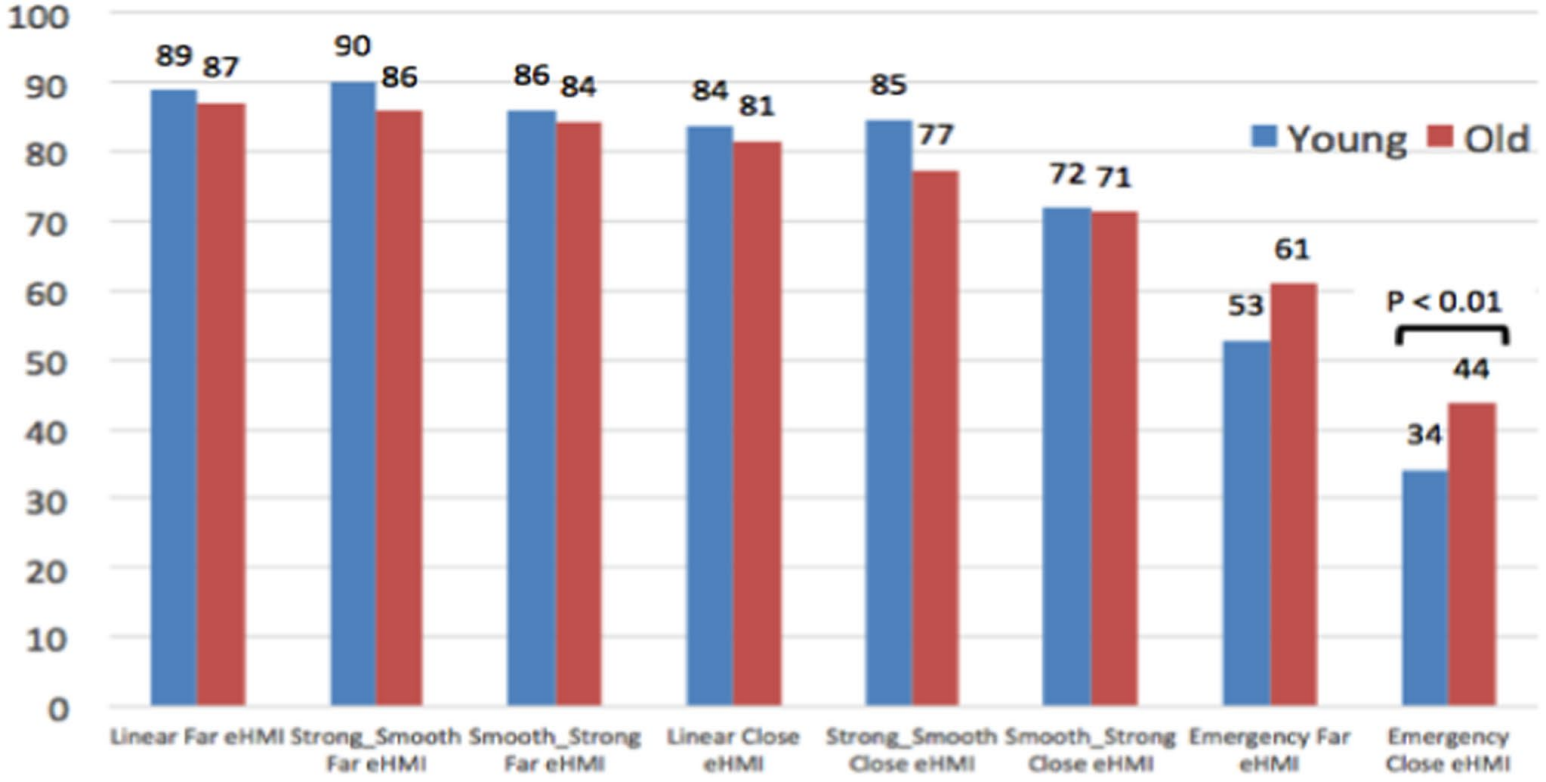
Safety assessment of AV (with eHMI) behavior to yield, as a pedestrian.

As a pedestrian (Figure 14), the only one significant difference observed between the two groups concerns the Close Emergency behavior, which is assessed as being safer by old participants. However, compared to the previous result presented in Figure 11, this inter-groups difference is here reduced by a third with the eHMI (10 points of difference with eHMI, against 15 without).

As a global result for this section dedicated to the perceived safety of AV behaviors to yield, it thus appears that the eHMI tends to reduce differences between young and old participants and to harmonize their safety assessments. With to the eHMI, elderly people seem more able to adequately perceive and assess the safety/dangerousness of AV braking maneuvers, and their safety judgments are at last close to those of young participants.

#### External HMI impact on AV acceptance

**Figure 15:**
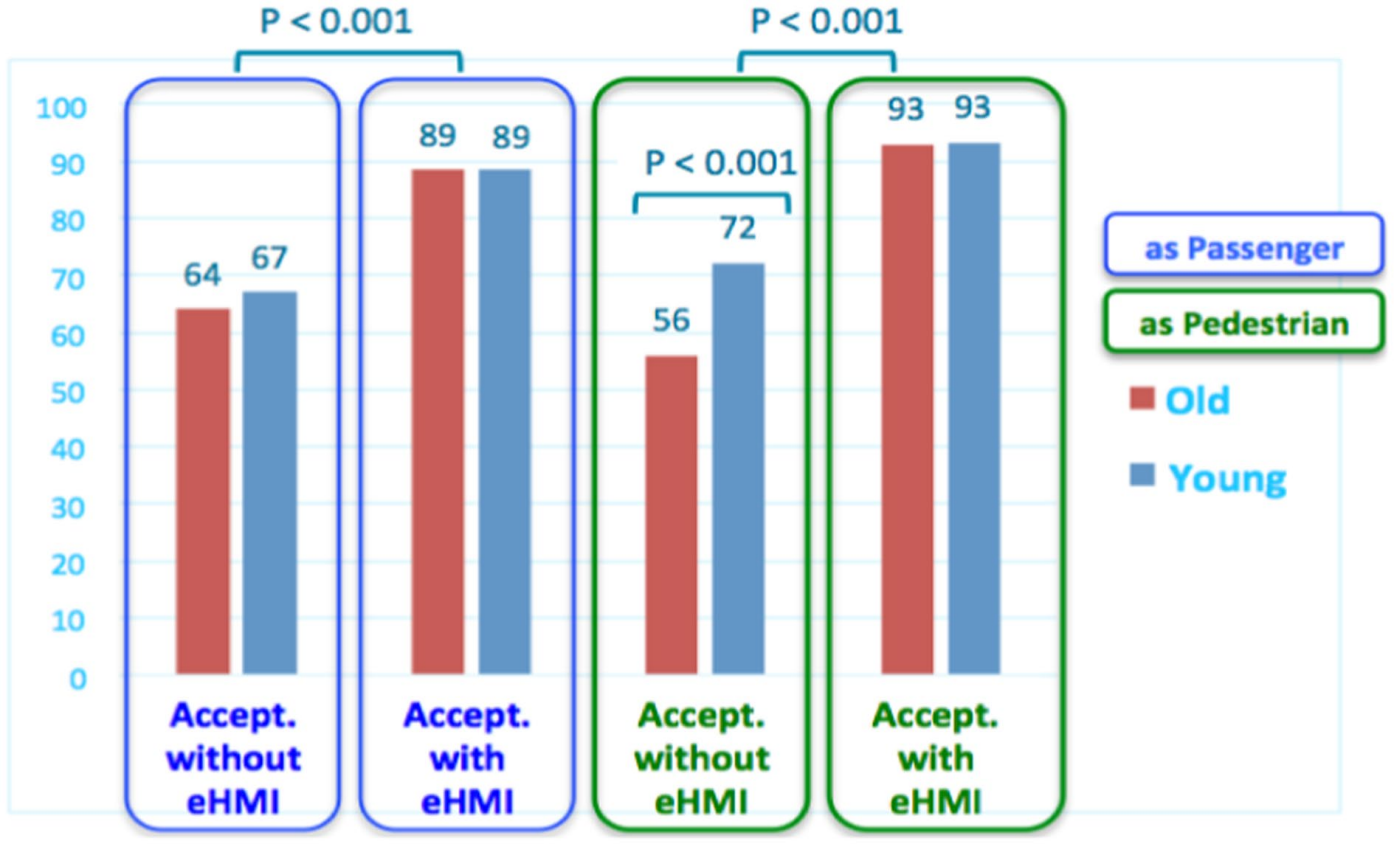
Effect of the eHMI on acceptance of AVs.

When considering the eHMI effect on AV acceptance, results presented in Figure 15 show that the eHMI significantly increases AV acceptance for both pedestrian and AV passenger, for all the participants (from 30 to 40% of acceptance gained).

This benefit is particularly important for elderly people as pedestrians. Indeed, their acceptance to interact with AVs without eHMI is relatively low compared to young participants (56 against 72) but, conversely, very high (and equivalent to young participants’ judgments) when the AV is equipped with the eHMI (93/100).

#### Evaluation of eHMI usefulness, intelligibility, and timing

After having experienced the eHMI, participants were invited to evaluate it regarding three complementary dimensions: (1) *Usefulness* to support their interactions with AVs, (2) *Intelligibility* of the information delivered, and (3) *Temporality* of the delivered pieces of information.

**Figure 16:**
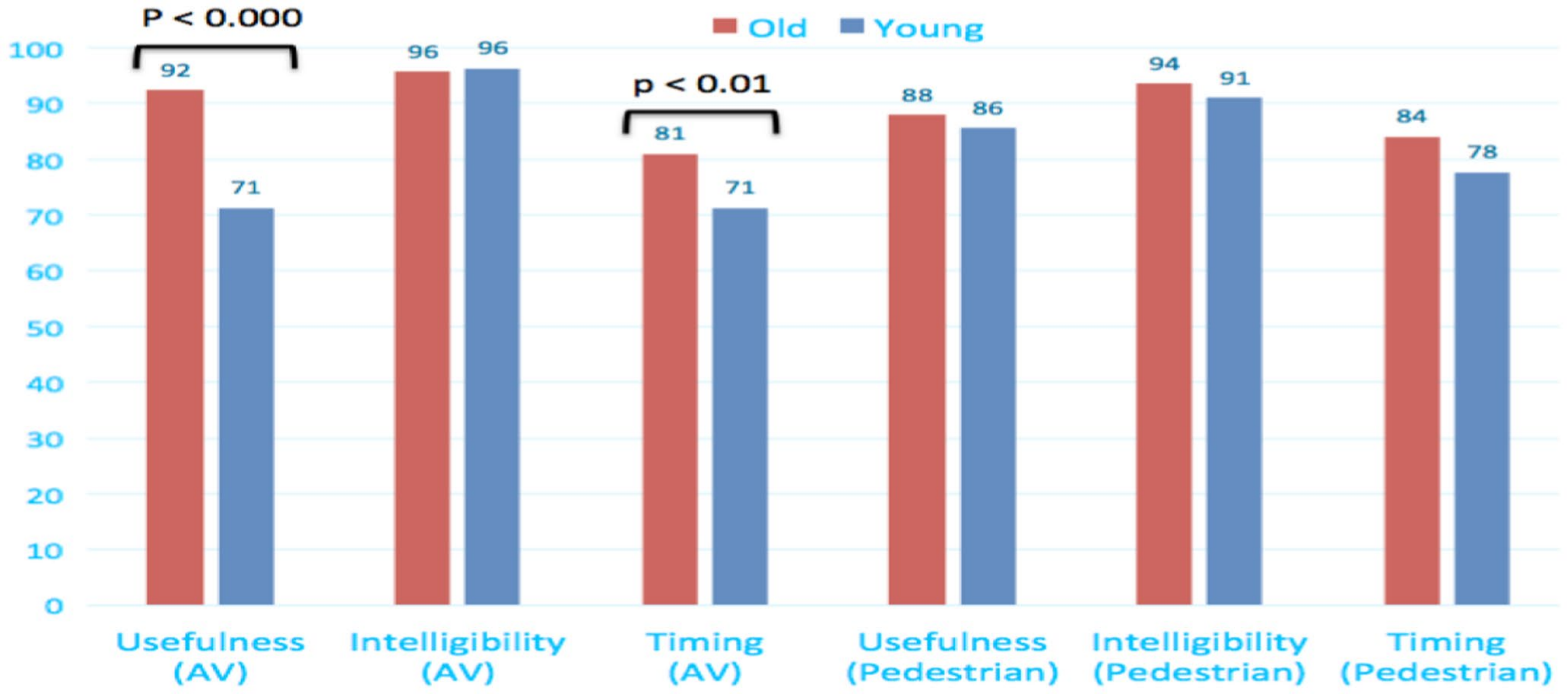
Evaluation of the eHMI usefulness, intelligibility and temporality.

Results presented in Figure 16 are positive for the two groups and for all these dimensions (scorings from 70 to 96/100). However, they are significantly higher for elderly people, compared to young, regarding eHMI *Usefulness* and *Timing* as AV passengers.

#### Automated vehicle safety and participants’ worry, before vs. after having experienced the eHMI

**Figure 17:**
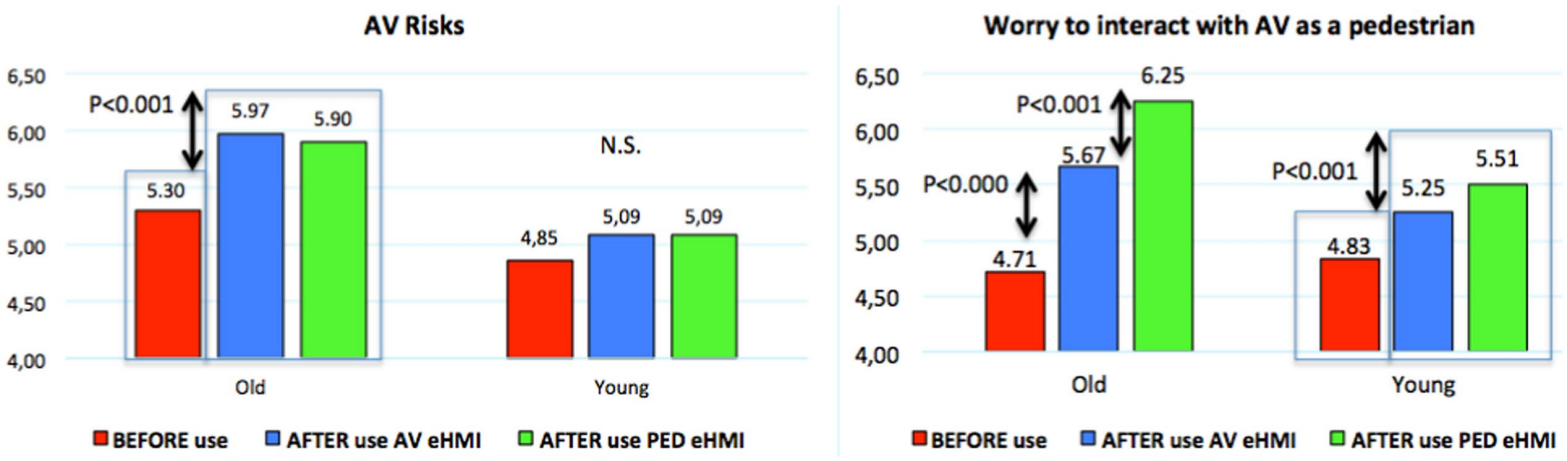
Automated vehicle safety questionnaire results (completed “before” and “after” the use of eHMI).

Finally, when considering the results collected from the AV Safety questionnaire administrated “Before” the experiment (i.e., *a priori* judgment) and then “After” having interacted with an Av equipped with the eHMI, two main results must be considered (Figure 17).

Regarding the 1st bloc focused on AV risks (i.e., “*AVs pose minimal risk to its driver and passengers*,” “*AVs pose minimal risk to other road users*” and “*AVs are safe*”), a significant (paired t-test) positive effect of the eHMI appears for the old participants, both as a pedestrian and as an AV passenger. Conversely, not any significant effect is found for the young participants.

For the 2nd bloc, dedicated to “worry to interact with an AV as a pedestrian” (i.e., “*I will cross the road in front of AVs,”* “*I have no concerns walking as usual if AVs would be on public roads*” and “*The prospect of interacting with AVs as a pedestrian appeals to me*”), results presented in Figure 17 are clear for all the participants: experiencing the eHMI significantly reduce their concerns related to interactions with AV. It is particularly the case for old participants as AV passenger (4.71 vs. 5.67/7) and, again more highly, as pedestrian (with a particularly high positive rating of 6.25/7).

## Discussion

The present study aimed at examining the judgments of two groups of participants [20 young mean-aged of 25.1 years old (50% female) and 20 old participants mean-aged of 67.5 years old (50% female)] when facing to eight AV behaviors to yield a pedestrian, in a non-protected area (i.e., without zebra crossing). All these yielding behaviors were experienced four times by each participant: as a “Pedestrian” and as an “AV Passenger,” and “Without” versus “With an eHMI.”

Initial research questions were about participants’ safety assessment of each one of these 8 AV yielding behaviors, and then about the evaluations of the benefits introduced by an external HMI to support them in this safety assessment.

Regarding the perceived safety of AV behaviors to yield without eHMI, our first hypothesis (H1) is confirmed for the stopping distance: “Far” stopping distances of 5 m (in front of the pedestrian) were assessed by all the participants as significantly safer than “Close” stopping distances of 2 m. Moreover, a common hierarchy (shared by all the participants) of the safety of the different braking maneuvers was established, ranging from Far Linear, Strong-Smooth, and Smooth-Strong behaviors (assessed as highly safe) to Emergency braking (including both Far and Close stopping distance) assessed as less safe. However, a set of significant differences was also observed between young and old participants. In a general way, young participants assessed AV behaviors similarly, whether as pedestrian or as AV passenger. Conversely, elderly participants have not the same way to perceive and assess the safety of the AV yielding behaviors from “the inside” and from “the outside” of the car: as pedestrians, they assess them significantly safer than as AV passengers. This is the case for both distances to stop (“Perceived safety of the AV behaviors according to the stopping distances”) as well as regarding the Emergency braking maneuvers simulating reactions of an AEB system facing a dangerous crossing of a pedestrian (“Perceived safety of AV behaviors to yield, according to participants’ age”). Because of their critical nature and their inappropriateness for the scenarios studied in this experiment, we expected a negative assessment from all participants about these emergency behaviors (whether associated with a Far or a Close stopping distance). This hypothesis is confirmed for the group of young participants and for elderly people as AV passengers. However, results obtained for old participants as pedestrians are different: compared to the young, their evaluations of the AEB braking were more positively evaluated, with a particularly high safety rating of 67/100 for the AEB Far stopping distance. According to the results of this study, it seems that old participants tend to overestimate the safety of AV behaviors as pedestrians. These results confirm our second hypothesis (H2) about the difficulties experienced by older people when they have to assess the speed of approaching vehicles and to identify a safe gap for road-crossing. As pedestrians, it seems that they have particular difficulties to visually perceive and adequately evaluate the abruptness of the AEB deceleration. Conversely, when on board the AV as passengers, they seem more aware about the inadequacy of such AEB emergency braking in order to yield a pedestrian who is quietly waiting on the pavement.

When considering results about benefits introduced by the eHMI, it firstly appears that this equipment may significantly reduce differences between young and old participants and tend to harmonize their assessments about the AV yielding behaviors: with this eHMI, elderly people are more able to adequately perceive and assess the safety/dangerousness of AV braking maneuvers, and their safety judgments become at last quite similar to those of young participants. It is particularly true as pedestrians. This is a first benefit of the eHMI in terms of road safety. Moreover, when considering participants’ assessments about AV “Acceptance,” it appears that a significant benefit is introduced by the eHMI, especially for old participants when interacting with AV as Pedestrian. Here again, the eHMI tends to harmonize the judgments of old and young participants. Finally, results based on the AV Safety questionnaire (completed “Before” and “After” having experienced the eHMI solution) show that the use of the eHMI significantly reduces participants’ concerns about their interactions with AVs as pedestrians, especially for elderly people.

## Conclusion

The experiment implemented in this study was designed to investigate the judgments of young and elderly participants when experiencing different AV yielding behaviors from two points of view: as a “Pedestrian” and as an “AV Passenger.” Results obtained allowed us to: (1) identify a set of AV braking strategies that are significantly assessed as safer from both the pedestrian and the AV passenger’s point of view, (2) detect some specific difficulties of elderly people, compared to young participants, to correctly perceive and assess the safety/dangerousness of AV braking behaviors, (3) confirm significant benefits introduced by the eHMI to support all the participants in their interactions with AVs, especially elderly people as pedestrians, and (4) show additional positive effects of the eHMI on participants’ judgments about “AV acceptance” and “feeling of safety” if they have to interact with AVs as external road users. The limitations of this study are that these results were obtained on a driving simulator and by using a Virtual Reality head-mounted display, and by considering only one situation of interaction. Some recent studies suggest that AV deceleration profiles can have different impacts on pedestrians’ experience based on the context (e.g., Dey et al., 2021). Thus, data collection in naturalistic conditions and covering a largest variety of road-crossing contexts will be required to generalize them. However, if confirmed in real traffic conditions, they may be of crucial importance for future road safety, i.e., when the AV will be introduced on public roads. Nowadays, elderly people are particularly vulnerable road users. If they effectively have more difficulties than other road users to perceive and assess adequately AV behaviors to yield, it should increase their risk of accident in the future. However, according to the results obtained in this experiment, an external HMI could actively support them in their future interactions with AVs, especially to support their perception and safety assessment when they would like to cross the road as a pedestrian in front of an approaching automated vehicle.

## Data availability statement

The original contributions presented in the study are included in the article/Supplementary material, further inquiries can be directed to the corresponding author.

## Ethics statement

The studies involving human participants were reviewed and approved by the Ethic committee of the University Gustave Eiffel. The patients/participants provided their written informed consent to participate in this study.

## Author contributions

TB: theoretical foundations and definition of research questions and hypotheses, conceptual design of the experiment, method definition, and data processing and analyses. SL: method definition and data collection. J-CB: theoretical foundations and definition of research questions and hypotheses, conceptual design of the experiment, and method definition. IH: data collection. BR: implementation of the driving scenario on the V-HCD simulator. All authors contributed to the article and approved the submitted version.

## Funding

This work was supported by the European Union’s Horizon 2020 Research and Innovation Program (i.e., SUaaVE project: SUpporting acceptance of automated VEhicles) under Grant 814999.

## Conflict of interest

J-CB is employed by the company ESI Group.

The remaining authors declare that the research was conducted in the absence of any financial relationships that could be construed as a potential conflict of interest.

## Publisher’s note

All claims expressed in this article are solely those of the authors and do not necessarily represent those of their affiliated organizations, or those of the publisher, the editors and the reviewers. Any product that may be evaluated in this article, or claim that may be made by its manufacturer, is not guaranteed or endorsed by the publisher.

## Footnotes

^1^Such a type of continuous analogue scales, like the one used in the GUI interface proposed by Chimi and Russell (2009), have been technically validated by these authors for collecting continuous data, and are now frequently used in the frame of web-based questionnaires or on-line surveys (e.g., Funke and Reips, 2012). In a comparative study, Yusoff and Mohd Janor (2014) also shown that 0–100 scales (p.13) "*performed better than data from 7-point Likert scales in terms of number of items per construct, factor loadings, squared multiple correlations, higher internal reliability, higher internal consistency of the items representing a construct, and higher percentage of variance explained by the items in a construct."* In addition, authors indicate (p. 14) that model based on 0–100 scales "*had higher ratio of df to number of parameters,"* provides *"more mathematical information to estimate model parameters* and *had higher convergent validity."*
